# Physiological reactivity to nonideographic virtual reality stimuli in veterans with and without PTSD

**DOI:** 10.1002/brb3.304

**Published:** 2014-12-23

**Authors:** Andrea K Webb, Ashley L Vincent, Alvin B Jin, Mark H Pollack

**Affiliations:** 1 The Charles Stark Draper LaboratoryCambridge, Massachusetts; 2 Department of Psychiatry, Rush University Medical CenterChicago, Illinois

**Keywords:** post-traumatic stress disorder, diagnosis, skin conductance, interbeat interval, classification accuracy

## Abstract

**Background:**

Post-traumatic stress disorder (PTSD) currently is diagnosed via clinical interview in which subjective self reports of traumatic events and associated experiences are discussed with a mental health professional. The reliability and validity of diagnoses can be improved with the use of objective physiological measures.

**Methods:**

In this study, physiological activity was recorded from 58 male veterans (PTSD Diagnosis *n *=* *16; Trauma Exposed/No PTSD Diagnosis: *n *=* *23; No Trauma/No PTSD Diagnosis: *n *=* *19) with and without PTSD and combat trauma exposure in response to emotionally evocative non-idiographic virtual reality stimuli.

**Results:**

Statistically significant differences among the Control, Trauma, and PTSD groups were present during the viewing of two virtual reality videos. Skin conductance and interbeat interval features were extracted for each of ten video events (five events of increasing severity per video). These features were submitted to three stepwise discriminant function analyses to assess classification accuracy for Control versus Trauma, Control versus PTSD, and Trauma versus PTSD pairings of participant groups. Leave-one-out cross-validation classification accuracy was between 71 and 94%.

**Conclusions:**

These results are promising and suggest the utility of objective physiological measures in assisting with PTSD diagnosis.

## Introduction

Post-traumatic stress disorder (PTSD) is a mental health disorder that can develop following a traumatic event. The traumatic event can involve actual or threatened death or harm to oneself or someone else. Symptoms of this disorder include re-experiencing of the event, numbing and avoidance, and hyperarousal (American Psychiatric Association 2000). PTSD can be associated with a variety of other disorders, such as anxiety, depression, and substance abuse, which can complicate both diagnosis and treatment. Although much publicized in the media as a disease affecting military veterans, PTSD affects civilians as well. Kessler et al. (2005) estimated a 6.8% lifetime prevalence of PTSD in the general population and Richardson et al. (2010) report prevalence rates for PTSD between 4 and 17% in veterans returning from Iraq. Ramchand et al. (2010) reported prevalence estimates between 5 and 20% in those not seeking treatment for PTSD in their review of studies conducted on veterans and nonmilitary personnel, whereas prevalence rates were approximately 50% amongst those who had sought treatment. Discrepancies in prevalence rates could be due to the self-report information needed to make a diagnosis. Some individuals may under report symptoms because of the stigma of having a mental health disorder, and others may over report symptoms to obtain medical benefits (Gates et al. 2012). It is well known that the costs of treating returning veterans are high. Some estimate the cost of treating Operation Enduring Freedom and Operation Iraqi Freedom veterans to approach $1 billion (Kilmer et al. 2011). Treatment costs, loss of productivity, and increased morbidity and mortality make it imperative that PTSD and its associated comorbidities be accurately diagnosed and effectively treated.

One of the primary challenges in accurately diagnosing PTSD is that diagnoses currently are made based upon the patient's subjective reports of his/her experiences related to the traumatic event as relayed during a clinical interview. As noted previously, subjects may under or over report symptoms, either consciously or inadvertently. More objective measures are needed to assist clinicians in diagnosing this disorder. The physiological consequences and symptoms of PTSD may provide such objective cues. Kardiner (1941) was one of the first to describe the psychophysiological assessment of PTSD. Kardiner noted that following combat, some patients experienced symptoms of tachycardia, muscle tension, startle, and hyper-responsivity to stimuli. Subsequent investigations have demonstrated physiological differences among those with and without PTSD, including changes in facial electromyography, heart rate, blood pressure, electrodermal reactivity, respiratory sinus arrhythmia, and eye movements (Blanchard et al. 1982; Blechert et al. 2007; Bryant et al. 1995;Gerardi et al. 1989; Pitman et al. 1990).

Past research has demonstrated that individuals with PTSD may show differential reactivity in response to specific, emotionally evocative cues. A number of studies have found that heart rate reactivity in response to standardized combat sound cues (e.g., gun fire, helicopters) can be used to discriminate those with and without PTSD (Blanchard et al. 1982, 1986, 1989; Pallmeyer et al. 1986). Idiographic cues specific to an individual's traumatic experience also are useful. Script-driven imagery is a technique often used in studies with idiographic cues. Greater skin conductance and heart rate responses have been elicited in those with PTSD as compared to those without using this technique (Keane et al. 1998; Orr et al. 1993). Other types of stimuli also have been utilized. McFall et al. (1990) found increased heart rate in Vietnam veterans with PTSD when watching videos of combat-related stress as compared to those without PTSD in comparison to noncombat-related videos. Taken together, these studies have demonstrated physiological differences in response to standardized and idiographic stimuli among those with and without PTSD. These findings are robust and consistent, as evidenced by a recent meta-analysis (Pole 2007), and suggest the utility of physiological reactivity in assisting with diagnosis.

Some research has examined differences in habituation for startle stimuli in those with and without PTSD. Orr et al. (1995) demonstrated a slower slope in recovery for electrodermal activity following startle cues in individuals with PTSD. Another study found no difference in the individual slopes of the startle responses, but did find a decreased level of habituation (Shalev et al. 1997). Jovanovic et al. (2009) did not find an exaggerated startle response but did find a difference in the degree of habituation to startle stimuli between those with and without PTSD. One of the goals of the present work was to assess differences in habituation to emotionally evocative stimuli in those with and without PTSD.

Virtual reality (VR) is a relatively new technology that could be used to present idiographic and standardized trauma cues and other stimuli. Many experts believe that VR may be used in the prevention, assessment, and eventual treatment of PTSD (Spira et al. 2010). VR is a multimodal technology that provides an immersive environment (Wood et al. 2010) that may be more useful than traditional cues and stimulus presentation methods alone. Several recent studies have found that VR exposure reduced PTSD scores (McLay et al. 2011, 2012; Reger et al. 2011). A recent study by Rothbaum et al. (2014) used the same VR platform and stimulus base as that used in this study and found improvements in PTSD symptoms. Indeed, VR technology has even been used on soldiers in active theater to enhance exposure therapy treatments (McLay et al. 2010). A recent review found that most treatment studies utilizing VR demonstrated improvement relative to wait list conditions, but that there was no difference between VR and traditional exposure therapies (Goncalves et al. 2012). Most agree that more treatment studies with VR are needed to assess its effectiveness compared to other treatment methods (Goncalves et al. 2012; Nelson 2012). Although additional research is needed, VR appears to be a promising method for treatment of PTSD. Given its success, additional efforts are underway to use the technology to assist in stress resilience training (Rizzo et al. 2013). Although this body of work is relatively new, it holds promise and supports the notion of using VR to assist in the diagnosis of PTSD.

Taken together, prior work has shown that objective physiological measures discriminate among those individuals with and without PTSD. Good results have been demonstrated with idiographic stimuli, particularly when utilized with a script-driven imagery paradigm. However, these methods may be difficult to apply to a point of care setting where there is limited time to solicit the idiographic cues and develop appropriate stimuli to elicit physiological reactivity. The goal of the present work was to assess the diagnostic utility of physiological features measured during the presentation of nonidiographic stimuli within a virtual reality context. It was predicted that there would be significant differences in the physiological features among those with and without trauma exposure and PTSD and that classification accuracies would be similar to those seen with idiographic stimuli. It is anticipated that such a methodology could eventually be used easily in a point of care setting to assist in the diagnosis process.

## Materials and Methods

### Participants

Participants were recruited using print and electronic advertisements (e.g., Craigslist), and flyers dispersed in the areas of Boston, MA and Tampa, FL. Of the 58 male participants who completed the protocol, 19 were in the No Trauma/No PTSD Diagnosis group, 23 were in the Trauma Exposed/No PTSD Diagnosis group, and 16 were in the PTSD Diagnosis group. There were no statistically significant site differences in any of the demographic variables, *P*s > 0.05. Demographic information is presented in Table 1.

**Table 1:** Demographic information

Variable	Control	Trauma	PTSD
Age	M* *=* *30.2 (SD* *= 7.92)	M* *=* *26.9 (SD* *= 3.61)	M* *=* *28.8 (SD* *= 4.29)
Years of Education	M* *=* *15.5 (SD* *= 2.04)	M* *=* *14.3 (SD* *= 1.33)	M* *=* *14.1 (SD* *= 1.67)
Employment			
Unemployed	3	6	9
Full-time	6	9	3
Part-time	6	5	2
Other	4	3	2
Marital Status			
Single	15	17	11
Married	2	5	2
Separated	0	1	0
Divorced	1	0	3
Widowed	1	0	0
Ethnicity			
Caucasian	15	18	14
Asian	1	1	0
African-American	2	1	1
Hispanic	1	3	1
Income			
<15,000	2	2	3
15,000–29,999	4	6	3
30,000–59,999	7	8	6
60,000–89,999	5	4	2
>90,000	1	3	2

### Apparatus

#### BIOPAC

The BIOPAC system (Goleta, CA) was used to collect respiration, skin conductance (SC), electrocardiograph (ECG), and finger pulse amplitude (FPA) measures. Respiration was recorded from a transducer secured around the upper chest with a Velcro strap. SC was recorded from disposable wet Ag-AgCl electrodes placed on the distal phalanges of the index and middle fingers of the nondominant hand. ECG was recorded from modified Lead II (lower left and upper right chest). FPA was recorded from the tip of the ring finger on the nondominant hand. Data were collected at 500 Hz.

#### eMagin Z800 3D visor

The eMagin Z800 3D head-mounted system (Bellevue, WA) provides a high-contrast virtual image and was used for presentation of the virtual reality videos. Participants wore head phones when using the 3D visor.

#### Virtual Iraq

Virtual Iraq software (Virtually Better, Decatur, GA) was used to create the virtual reality videos. Virtual Iraq is typically used in a therapeutic context in which the client is allowed to navigate through the virtual scene using a game controller. In this study, the software was used to create two combat-related videos in which stimuli of increasing severity were programmed to appear. One video was a humvee driving scene and the other video was designed to emulate a foot patrol in a city setting. The five stimuli in each video were comparable and were an aircraft flying overhead, a mortar explosion, an improvised explosive device (IED), an attack resulting in an explosion, and an attack by an insurgent. The five events occurred at approximately 30, 75, 120, 165, and 210 s after the start of the video.

### Procedure

#### Research session

Data collection occurred during a single session that lasted between 3 and 4 h. Participants provided written informed consent prior to beginning the study. The study was approved by the New England Institutional Review Board. Participants earned $25 per hour.

After providing informed consent, participants completed a demographic information questionnaire, and provided information regarding medical history, medications, and substance use. Additionally, participants completed both the State Trait Anxiety Inventory (STAI) (Spielberger et al. 1983) and the Positive and Negative Affect Schedule general and present forms (PANAS) (Watson et al. 1988) to determine baseline measures of anxiety and affect. All participants also completed the Traumatic Events Questionnaire (TEQ, Revised 7-2004) (Vrana and Lauterbach 1994) and the Structured Clinical Interview for DSM-IV-TR Axis I Disorders (SCID-I/P), Post-traumatic Stress section (First et al. 2002). The SCID was used to confirm current diagnosis of PTSD and no lifetime history of PTSD in the Trauma and Control groups. Those who appeared to be in either the PTSD Diagnosis (PTSD) or Trauma Exposed/No PTSD Diagnosis (Trauma) groups also completed the Clinician-Administered PTSD Scale (CAPS) (Blake et al. 1995) for confirmation of and more detail regarding symptoms. Total severity lifetime scores for the CAPS for those in the PTSD group ranged between 48 and 122 (M =* *77.13, SD* *= 20.16). Scores for those in the Trauma group ranged between 0 and 25 (M* *=* *3.17, SD* *= 7.66).

Physiological data collection began with a 10 min baseline rest period, followed by eight audio startle stimuli. Participants then experienced 24 emotionally evocative images from the International Affective Picture System (IAPS) (Lang et al. 2008), 24 emotionally evocative sounds from the International Affective Digitized Sound system (IADS) (Bradley and Lang 1999), and two virtual reality (VR) videos. Order of the emotionally evocative stimuli (IAPS, IADS, and VR videos) was counterbalanced and randomized across participants.

Upon completion of all stimulus presentation trials, participants were asked to again complete the STAI (Y1 – current) and PANAS (present) as well as a stimulus reaction questionnaire allowing them to provide qualitative responses about the stimuli. Participants were given the opportunity to ask questions or provide feedback, paid, thanked, and released.

### Data analysis approach

The analyses presented here focus on SC and interbeat interval (IBI) for the VR portions of the protocol. CPSLAB (Scientific Assessment Technologies, Salt Lake City, UT) was used to visually examine, edit and generate response curve features for the physiological data. Artifacts were visually identified and removed from the data via the interpolation function in CPSLAB.

#### Response curves

Response curves were computed for each signal. For the SC data, the response curve was defined by the sequence of values. For the ECG data, R-peaks were identified and used to create an IBI waveform. For each VR video, a response curve was computed for each of the five video events. The response curve began at the onset of the event and ended 20 s later. This time window was chosen to capture the physiological response and return to baseline for each event.

#### Feature extraction

In contrast to previous work, a variety of features were extracted from each signal to provide a richer characterization of the response to each video event. The features selected allow for an assessment of differences across stimuli and groups in response magnitude, timing, and variability. The following features were extracted from the ten VR video event response curves for both SC and IBI using the CPSLAB feature library and extraction function:

*Peak amplitude* was computed by identifying high and low points on the response curve. Low points were identified as changes from negative or zero slope to positive slope, and high points were identified as changes from positive slope to zero or negative slope. The difference between each low point and every succeeding high point was computed. Peak amplitude was the greatest difference.
*Area to full recovery* was the area under the response curve from response onset to the point of full recovery. If the point of full recovery was not reached within the extraction window, the last data point within the window was used to define the point of full recovery.
*Full recovery time* was computed by subtracting the time at which the peak amplitude occurred from the time at which the signal recovered to baseline. If the signal did not return to baseline within the extraction window, the last data point within the window was used to define the full recovery time.
*Latency to first low point* was the time between stimulus onset and the onset of the physiological response.
*Standard deviation* was the standard deviation of samples that defined the response curve.
*Phasic level* was the average of the data points within the response window.

#### Within-subject standardization

For each feature, a measurement was obtained for each VR video event. The 10 measurements were converted to z-scores for each subject.

#### Difference scores

Difference scores were calculated by subtracting feature values for SC and IBI for each event in video 1 from scores for each event in video 2, such that negative difference scores indicate a decrease in the feature value from video 1 to video 2. These differences were computed for each of the features described above. Statistical analyses were conducted with SPSS-21 (IBM, Armonk, NY).

## Results

An alpha level of 0.05 was used. Bonferroni-corrected *P*-values were used for post hoc comparisons. Repeated measures analysis of variance (RMANOVA) was conducted to assess site (Boston, Tampa) differences in the five events for each feature. None of the main effects for site were statistically significant, *P *>* *0.05, therefore site was not included as a factor in the analyses presented below.

### Feature analysis

#### Skin conductance

Means and standard deviations for feature value differences across the two videos for each event are presented in Table 2. A 3 (Group: Control, Trauma, PTSD) ×5 (Video event) RMANOVA was conducted to examine changes in difference scores across the five video events. Of most relevance are the main effect of Group and the Event x Group interaction, which are reported for each feature. A significant main effect of Group was found (*F*_2,53_ = 4.38, *P *=* *0.02, 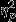 *= *0.14) for SC amplitude. Pairwise comparisons revealed significantly greater decreases in the Control group as compared to the PTSD group. Significant simple effects of group were found within Event 1 (Aircraft) and Event 3 (IED; *F*_2,53_ = 4.96, *P *=* *0.01, 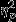 = 0.16 and *F*_2,53_ = 4.92, *P *=* *0.01, 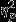 = 0.16, respectively) and pairwise comparisons revealed the same trend of significantly greater decreases in the Control group as compared to the PTSD group for both events.

**Table 2:** Means (SD) for VR SC feature differences in each event

		VR video event differences				
Feature	Group	1 Aircraft	2 Mortar	3 IED	4 Attack	5 Insurgent
Amplitude	Control	−14.05 (8.68)	−11.73 (13.00)	−16.39 (7.85)	−9.66 (9.44)	−10.04 (10.90)
	Trauma	−9.86 (13.48)	−12.61 (12.38)	−9.48 (13.64)	−3.84 (12.24)	−3.46 (15.08)
	PTSD	0.86 (19.21)	−3.21 (15.54)	−1.77 (17.88)	−3.13 (16.33)	−4.32 (16.50)
Area to full recovery	Control	−13.77 (10.43)	−11.18 (14.99)	−14.29 (7.25)	−10.18 (11.25)	−10.40 (11.99)
	Trauma	−10.49 (14.98)	−13.38 (11.31)	−8.63 (13.91)	−4.99 (13.82)	−4.89 (14.56)
	PTSD	2.57 (19.18)	−3.16 (16.13)	−1.11 (18.60)	−2.39 (16.30)	−4.14 (16.69)
Full recovery time	Control	8.95 (11.15)	2.28 (18.81)	4.86 (11.71)	1.42 (11.08)	−2.94 (10.34)
	Trauma	3.28 (13.67)	−4.34 (15.30)	0.00 (11.44)	−0.06 (13.59)	−1.46 (15.78)
	PTSD	4.47 (12.55)	−1.56 (15.82)	4.60 (10.36)	−1.15 (8.89)	1.84 (10.67)
Standard deviation	Control	−10.58 (10.54)	−9.23 (12.63)	−12.71 (12.64)	−0.24 (11.15)	−7.09 (12.46)
	Trauma	−5.51 (16.04)	−8.01 (13.10)	−5.01 (14.49)	4.75 (10.71)	1.21 (13.99)
	PTSD	−1.36 (17.14)	−3.65 (13.76)	0.52 (13.96)	−4.44 (13.93)	−3.94 (12.76)
Level	Control	−14.93 (10.61)	−13.30 (13.79)	−14.73 (6.68)	−10.08 (11.36)	−9.96 (12.61)
	Trauma	−10.47 (14.96)	−13.39 (11.30)	−8.63 (13.91)	−5.01 (13.81)	−4.89 (14.55)
	PTSD	2.57 (19.19)	−3.13 (17.00)	2.03 (17.10)	−2.05 (16.42)	−5.42 (14.06)
Latency to first low point	Control	−1.55 (16.19)	−1.88 (8.61)	−2.41 (11.63)	−2.38 (15.23)	−1.53 (14.91)
	Trauma	3.47 (13.60)	−3.54 (9.96)	−2.27 (14.10)	−6.46 (17.10)	0.91 (12.55)
	PTSD	3.28 (11.79)	0.19 (9.86)	−2.62 (14.08)	0.36 (15.84)	−0.99 (13.46)

Similarly for area to full recovery, a significant main effect of Group was found (*F*_2,53_ = 3.90, *P *=* *0.03, 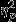 = 0.13). Pairwise comparisons revealed significantly greater decreases in SC area to full recovery in the Control group as compared to the PTSD group. Significant simple effects of group were found within Event 1 (Aircraft) and Event 3 (IED; *F*_2,53_ = 5.53, *P *=* *0.01, 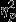 = 0.17 and *F*_2,53_ = 3.86, *P *=* *0.03, respectively) and pairwise comparisons revealed the same trend of significantly greater decreases in the Control group as compared to the PTSD group for both events.

The main effect of Group and the Event × Group interaction were not significant for full recovery time.

For standard deviation, a marginally significant main effect of Group was found (*F*_2,53_ = 3.29, *P *=* *0.05, 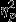 = 0.11). Pairwise comparisons revealed greater decreases in SC standard deviation in the Control group as compared to the Trauma group.

A significant main effect of Group was found for level (*F*_2,53_ = 4.92, *P *=* *0.01, 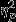 = 0.16). Similar to the pattern present for other features, pairwise comparisons revealed significantly greater decreases in the Control group as compared to the PTSD group. Significant simple effects of group were found within Event 1 and Event 3 (*F*_2,53_ = 6.22, *P *<* *0.01, 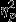 = 0.19 and *F*_2,53_ = 6.97, *P *<* *0.01, 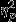 = 0.21, respectively) and pairwise comparisons revealed the same trend of significantly greater decreases in the Control group as compared to the PTSD group for both events. Similarly, the trauma group showed greater decreases than the PTSD group for both events.

There were no statistically significant effects for latency to first low point.

Taken together, the results of the RMANOVAs for skin conductance demonstrate that those in the PTSD group did not habituate while watching the two VR videos, whereas the Control group did, and that these effects were most pronounced for the first and third events in the videos.

#### Interbeat interval

For time to full recovery, the event by group interaction was marginally significant, *F*_8,208_ = 1.75, *P *=* *0.09, 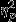 = 0.06. Significant simple effects of group were found within Event 3, *F*_2,52_ = 5.38, *P *=* *0.01, 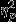 = 0.17. The trauma group differed significantly from both the Control and PTSD groups (M_Control_ = −0.02, SD_Control_ = 11.92 and M_Trauma_ = 11.53, SD_Trauma_ = 15.27; M_PTSD_ = −1.61, SD_PTSD_ = 13.18).

For latency to the first low point, the interaction between event and group was marginally significant, *F*_4,208_ = 1.82, *P *=* *0.08, 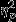 = 0.07. Significant simple effects of group were found within Event 1, *F*_2,52_ = 3.90, *P *=* *0.03, 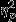 = 0.13. The control group (M* *=* *−9.71, SD* *= 13.86) showed greater decreases than both the trauma group (M* *=* *1.53, SD* *= 15.17) and the PTSD group (M* *=* *2.29, SD* *= 13.14), *P*s = 0.05 and 0.06, respectively.

There were no statistically significant effects for amplitude, area to full recovery, standard deviation, or level.

### Classification accuracy

The IBI and SC features for each of the ten VR video events (five from each of the two videos) were submitted to stepwise discriminant function analysis (DFA) to assess classification accuracy. Three DFAs were performed to assess which variables were selected for Control versus Trauma, Control versus PTSD, and Trauma versus PTSD pairings of the group variable. Notably, DFA selected different variables for each of these analyses. For the Control versus Trauma analysis, SC amplitude for Event 3, IBI full recovery time for Event 3, and IBI latency for Event 1 were selected. For the Control versus PTSD analysis, SC level for Event 3 and IBI amplitude for Event 4 were selected. For the Trauma versus PTSD analysis, SC amplitude for Event 5, SC level for Event 1, IBI amplitude for the Event 1, IBI full recovery time for Event 3, IBI latency for Events 4 and 5, IBI level for Event 1, and IBI standard deviation for Event 5 were selected. For the PTSD and Trauma analysis, classification accuracy was 100.0 and 95.5% for the two groups, respectively, and leave-one-out cross-validated classification accuracy was 93.8 and 81.8%, respectively. For the Control and PTSD analysis, performance was identical for the original and cross-validated analyses. Of the Control subjects, 88.2% were correctly classified and 75.0% of the PTSD subjects were correctly classified. For the Control and Trauma analysis, 76.5 and 77.3% were correctly classified. In the cross-validated analysis, 70.6% of the Control subjects and 72.7% of the Trauma subjects were correctly classified.

The intercorrelation matrix for the variables selected by the DFA is presented in Table 3. Although some of the features were not statistically significant in RMANOVA, they did provide some discriminatory power, as indicated by the DFAs. Table 3 also contains point-biserial correlations for the features and the three group status variables.

**Table 3:** Intercorrelations among variables selected by DFA

						SC				IBI							
		Feature	Trauma (0), PTSD (1)	Control (0), PTSD (1)	Control (0), Trauma (1)	1	2	3	4	5	6	7	8	9	10	11	12
SC	1	AMP, Event 3	0.24	0.49**	0.30	–											
	2	AMP, Event 5	−0.03	0.21	0.24	0.34**	–										
	3	LEV, Event 1	0.37*	0.51**	0.17	0.48**	0.19	–									
	4	LEV, Event 3	0.33*	0.56**	0.27	0.91**	0.47**	0.58**	–								
IBI	5	AMP, Event 1	−0.31	−0.14	0.19	−0.06	−0.05	0.02	−0.04	–							
	6	AMP, Event 4	0.02	−0.19	−0.23	0.20	−0.11	0.07	0.14	0.11	–						
	7	FRT, Event 3	−0.42**	−0.07	0.39*	−0.11	−0.15	−0.01	−0.11	0.04	−0.11	–					
	8	LA1, Event 1	0.03	0.42*	0.37*	−0.07	−0.04	0.08	−0.06	−0.08	0.02	−0.09	–				
	9	LA1, Event 4	0.32*	0.25	−0.10	0.11	0.17	−0.10	0.14	−0.03	0.03	0.08	0.05	–			
	10	LA1, Event 5	0.20	0.13	−0.06	−0.01	−0.01	0.02	0.02	0.05	−0.02	0.17	0.08	0.12	–		
	11	LEV, Event 1	0.13	0.07	−0.05	0.13	0.30*	0.15	0.12	−0.03	−0.19	0.04	−0.30*	0.05	0.06	–	
	12	STD, Event 5	−0.20	0.07	0.25	0.30*	0.00	0.25	0.22	−0.15	−0.16	0.01	−0.10	−0.17	−0.03	0.23	–

AMP, amplitude; LEV,level; FRT, full recovery time; LA1, latency to first low point; STD, standard deviation.

* *P *<* *0.05, **P <* *0.01.

## Discussion

The features extracted from skin conductance and interbeat interval waveforms obtained in response to nonidiographic emotionally evocative virtual reality stimuli discriminated well among those with and without trauma and with and without PTSD. Classification accuracies were well above chance levels for all analyses. Notably, optimal features selected by each DFA differed for each pairwise group analysis. This suggests that a number of different diagnostic algorithms may be needed for optimal performance in point of care settings. Although the results are promising, false positives and false negatives were present. Additional work is needed to understand the characteristics of those who are incorrectly classified, particularly in a larger and more heterogeneous sample. Such information could be used to tailor algorithms for a more individualized approach. Recent literature reviews have highlighted the complexities and challenges in understanding the biology of PTSD and the search for biomarkers of PTSD (Pitman et al. 2012; Zoladz and Diamond 2013). Although the psychophysiological indicators assessed in this study are promising, it is clear that multiple indicators will be needed to fully characterize this disorder to assist in diagnosis.

Consistent with prior work (Blanchard et al. 1986; Carson et al. 2000; Keane et al. 1998), there were differences among those with and without PTSD in cardiac and electrodermal activity in this study. The nonideographic stimuli presented in the virtual reality videos were effective in eliciting physiological responses in all three of the groups. Those in the PTSD group tended to have skin conductance responses that did not habituate across presentation of the two videos, whereas the trauma and control groups did tend to show habituation. This trend was present for most of the skin conductance features that were examined and was most pronounced for the first and third events presented in the videos. This lack of habituation in the PTSD group supports prior work and what is known about the physiological consequences of PTSD. Fewer interbeat interval features demonstrated statistical significance in RMANOVA but did provide discriminatory power in the DFA.

The classification accuracies achieved in this study are consistent with those found in other work in which reactivity was elicited with trauma-related cues. Orr (1997) noted that psychophysiological assessments have produced specificity values between 80 and 100% and sensitivity values between 60 and 90%. Although the VR stimuli could be viewed as more complex than stimuli used in past work (e.g., static images, script-driven imagery), their success in eliciting reactivity bodes well for a technology that is increasing in use in the treatment domain and that has promise for the diagnostic domain as well. As noted previously, VR is a more immersive technology than what is typically used to elicit reactivity, and it may be more realistic and widely accepted by a generation already familiar with and comfortable with such technology. There may be barriers to implementation of VR in point of care settings that will need to be addressed as the research and technology advance.

Many studies on the psychophysiology of PTSD have focused on different types of features than those presented here. Rather than focusing on features such as heart rate change or skin conductance level, as has been done in previous studies, this work utilized an approach in which multiple features are extracted from event-related waveforms to provide a richer characterization of the physiological response. Although some of the features were correlated with one another (see Table 3), many of them were not and provided unique discriminatory information. This approach allows for the extraction of many features for each event, but it can be problematic in terms of the robustness of the discriminant functions, particularly with a small sample like this one. Replication with a larger sample is needed.

A few limitations of this study must be noted. Participants in this study were male veterans recruited primarily via Craigslist and other media sources. This approach differs from other studies in which veterans are recruited directly from Veteran's Administration (VA) hospitals. It is possible that the current sample differs in some way from samples recruited from VA hospitals. Future work should be done with female veterans and civilians to ascertain the generalizability of these findings. Finally, participants in this study were generally free of comorbid mental health conditions. Given that PTSD is often comorbid with a variety of other disorders, future work should be carried out with a more heterogeneous sample to ascertain the generalizability of these findings. It may be that additional physiological features and stimuli are necessary to discriminate among those with different combinations of comorbid disorders and/or different combinations of symptoms. As noted previously, PTSD is a complex disorder and many indicators will likely be needed to assist with prevention, diagnosis, and treatment.
